# Identification of two mutant *JASON-RELATED* genes associated with unreduced pollen production in potato

**DOI:** 10.1007/s00122-024-04563-7

**Published:** 2024-03-12

**Authors:** Corentin R. Clot, Lea Vexler, Maria de La O Leyva-Perez, Peter M. Bourke, Christel J. M. Engelen, Ronald C. B. Hutten, José van de Belt, Erik Wijnker, Dan Milbourne, Richard G. F. Visser, Martina Juranić, Herman J. van Eck

**Affiliations:** 1 Plant Breeding, Wageningen University and Research, Po Box 386, 6700 AJ Wageningen, The Netherlands; 2 Laboratory of Genetics, Wageningen University and Research, Po Box 16, 6700 AA Wageningen, The Netherlands; 3 Teagasc, Crops Research, Oak Park, Carlow, R93 XE12 Ireland

## Abstract

**Key message:**

Multiple QTLs control unreduced pollen production in potato. Two major-effect QTLs co-locate with mutant alleles of genes with homology to AtJAS, a known regulator of meiotic spindle orientation.

**Abstract:**

In diploid potato the production of unreduced gametes with a diploid (2*n*) rather than a haploid (*n*) number of chromosomes has been widely reported. Besides their evolutionary important role in sexual polyploidisation, unreduced gametes also have a practical value for potato breeding as a bridge between diploid and tetraploid germplasm. Although early articles argued for a monogenic recessive inheritance, the genetic basis of unreduced pollen production in potato has remained elusive. Here, three diploid full-sib populations were genotyped with an amplicon sequencing approach and phenotyped for unreduced pollen production across two growing seasons. We identified two minor-effect and three major-effect QTLs regulating this trait. The two QTLs with the largest effect displayed a recessive inheritance and an additive interaction. Both QTLs co-localised with genes encoding for putative *AtJAS* homologs, a key regulator of meiosis II spindle orientation in *Arabidopsis thaliana*. The function of these candidate genes is consistent with the cytological phenotype of mis-oriented metaphase II plates observed in the parental clones. The alleles associated with elevated levels of unreduced pollen showed deleterious mutation events: an exonic transposon insert causing a premature stop, and an amino acid change within a highly conserved domain. Taken together, our findings shed light on the natural variation underlying unreduced pollen production in potato and will facilitate interploidy breeding by enabling marker-assisted selection for this trait.

**Supplementary Information:**

The online version contains supplementary material available at 10.1007/s00122-024-04563-7.

## Introduction

Unreduced gametes, also known as 2*n* gametes, are gametes with a diploid (2*n*) rather than a haploid (*n*) number of chromosomes. Sexual polyploidisation through unreduced gametes is proposed as a major route to polyploid formation (Ramsey and Schemske 1998; Tayalé and Parisod 2013). Polyploids are common among flowering plants and overrepresented in crops (Otto and Whitton 2000; Salman-Minkov et al. 2016). It is suggested that polyploids were selected pre- or post-domestication because of their greater phenotypical plasticity, larger organ size and reproductive isolation from wild-relatives (Bretagnolle and Thompson 1995; Dempewolf et al. 2012; Sattler et al. 2016). Unreduced gametes are also an important tool for plant breeders who used them to link germplasm of different ploidy levels and for the induction of new polyploids (Ramanna and Jacobsen 2003; Younis et al. 2014). Unreduced gametes generally result from an altered meiotic process affecting either chromosomal segregation or cell division. The most widely reported mechanism of male meiotic restitution is the misorientation of metaphase II (MII) plate and spindles also known as parallel spindles (Bretagnolle and Thompson 1995). In some species, simultaneous cytokinesis, the misorientation of MII plates can lead to the production of unreduced gametes that contain homologous chromosomes and are genetically equivalent to a First Division Restitution (FDR). Alternatively, unreduced gametes obtained from an omission of the second division are composed of sister chromatids and are genetically equivalent to a Second Division Restitution (SDR). Those restitution mechanisms can be caused by a combination of environmental and genetic factors (De Storme and Geelen 2013, 2014).

Here we are interested in the genetic basis of unreduced pollen production in potato (*Solanum tuberosum*). The production of unreduced pollen via misorientation of MII plates and spindles has been commonly recorded among tuber-bearing *Solanum* species (Watanabe and Peloquin 1989). The inheritance of this meiotic restitution mechanism and two independent types of premature cytokinesis were described as independent monogenic recessively inherited traits (Mok and Peloquin 1975a, b). The subsequent re-investigation of those cytological mechanisms by Ramanna (1979) drew a more complex picture of the situation where the co-occurrence of various restitution mechanisms in a single clone could not be excluded. Likewise, analysis of the genetic control of unreduced pollen formation during cycles of recurrent selection and in diallel crosses suggested a more complex polygenic inheritance (Ortiz and Peloquin 1992; Dongyu et al. 1995). While unreduced gametes have been successfully used to introgress resistance genes from diploid wild relatives of potato to tetraploid cultivars (Ortiz et al. 1994; Capo et al. 2002; Zimnoch-Guzowska and Flis 2021), genetic loci involved in their production have never been identified. Meanwhile, the study of *Arabidopsis thaliana* mutants uncovered several meiotic pathways regulating unreduced gamete formation (De Storme and Geelen 2013). Key players in those pathways include *AtPS1* (*A. thaliana Parallel Spindle 1*) and *AtJAS* (*A. thaliana JASON*) which are known to regulate the orientation of the MII plate and spindle. Knock-out of either of those genes results in the formation of FDR unreduced pollen (D’Erfurth et al. 2008; De Storme and Geelen 2011). On the other hand, loss of function of *TAM* (*TARDY ASYNCHRONOUS MEIOSIS*) or *OSD1* (*OMISSION OF SECOND DIVISION*) leads to the failure of entering meiosis II and to the production of SDR dyads (d’Erfurth et al. 2009, 2010).

Through a comprehensive study spanning multiple years and several populations, we discovered a total of five QTLs regulating unreduced pollen production diploid potato. Two major-effect QTLs co-localised with genes sharing homology with *AtJAS*. We also identified deleterious sequence variants in those genes, namely a transposon insertion within an exon and an amino acid substitution within a domain that is highly conserved across vascular plants. Besides facilitating interploidy breeding for potato, our findings provide the first examples of natural genetic variation associated with unreduced pollen production.

## Materials and methods

### Plant material

In this study, we used three diploid *S. tuberosum* mapping populations: FRW19-112, IVP16-560, and CE-XW, which resulted from the crosses IVP92-057-3 × IVP92-030-14, RH89-039-16 × IVP10-281-1, and C (USW 5337.3) × E (77.2102.37), respectively. Detailed information on these clones and their related pedigrees is provided in Figure S1. In previous growing seasons the parental clones RH89-039-16, IVP92-057-3, C, and E produced unreduced pollen, which was confirmed to be functional through successful 4x × 2x crosses (Hutten et al. 1994; Park et al. 2007). The three mapping populations have a shared ancestry, as C is a grand-parent of RH89-039-16 and both C and E are grand-parents of IVP92-057-3. In 2019, 124 descendants from population IVP16-560 were grown from seed tubers seeds in five litre pots and under 16 h of light in an greenhouse compartment with drip irrigation and an evaporative cooling system. In 2020, this population was extended to 191 clones grown from seed tubers in a single row field (51°57′05.7″N; 5°38′02.5″E) planted the 17th of April. In 2020, 94 descendants from population FRW19-112 were grown from true potato seeds in five litre pots and under 16 h of light in an conditions previously described. In 2020, 225 CE-XW seedlings grown under greenhouse conditions as described in Clot et al. (2023b) were phenotyped for unreduced pollen production. In 2022, this population was extended to 268 clones grown from seed tubers in 19-cm pots at ambient temperatures and under 16 h of light.

### Analysis of male meiosis

To observe the male meiosis progression and chromosomal behaviour of the parental clones of population FRW19-112 and IVP16-560, we fixed floral buds of 3- to 4-mm parental clones in Carnoy solution (3:1 EtOH (99.8%): glacial acetic acid). We refreshed the Carnoy solution during the harvest day and kept overnight at 4 °C. Next, we washed the buds twice with 70% EtOH and stored them at 4 °C and followed the chromosome spread technique described by Jones and Heslop-Harrison (1996). After rinsing the fixated buds in water, we dissected the anthers and washed them a second time in water. The anthers were then incubated at 37 °C for 1.2 h in a 1:1 mixture of pectolytic enzymes and 10 mM citrate buffer (pH 4.39). After enzymatic digestion, we macerated a single anther on a slide in a small drop of 60% acetic acid and stirred it gently on a hotplate at 55 °C for 1 min. We then flooded the slide with freshly made Carnoy solution and dried it on a hotplate for 2 min. Finally, we stained the slides with 12 mL of DAPI diluted in Vectashield (300 ng/µL) and mounted them with a coverslip. We kept the slides at 4 °C until we observed them using a Axio Imager Z2 microscope equipped with an external light source (X-cite series 120 EXFO), a 120W high-pressure metal halide lamp, a DAPI reflector, and an Axiocam 506 camera. We conducted a comparative analysis of progression of meiosis by examining five anthers per clone spanning stages from pachytene to tetrad. For each anther, we recorded the meiotic stage of at least 200 and up to 250 meiocytes. Similarly, we compared the proportion of different MII plate orientations based on at least three and up to five anthers per clone. For each anther, 20–70 meiocytes at metaphase II were observed and classified as having normal, parallel, or fused MII plates following Ramanna (1979). For each orientation, we performed a statistical comparison of clone-specific medians using the nonparametric Kruskal–Wallis test and the Conover-Inman post hoc test (*α* = 0.05) corrected for multiple comparison with Holm procedure (Conover and Iman 1979; Holm 1979).

### Phenotyping unreduced pollen production

For each growing season, we collected up to four pollen samples per clone on different days throughout the flowering period. The samples were composed of bulked pollen extracted using a vibrator pin (modified electric toothbrush) from two flowers at anthesis with freshly opened anther pores. We stained the pollen samples using a simplified version of Alexander staining (Peterson et al. 2010) and examined them under bright field using a Axiophot Zeiss microscope equipped with a Neofluar 10x/0.30 lens. For each pollen sample, we photographed four field views using a Zeiss Axiocam ICc 5 colour camera and Zen 2.3 lite software. We considered these four images as a single observation and analysed them using Fiji (Schindelin et al. 2012) and a modified version of the macro developed by Tello et al. (2018) (File S1) to determine pollen diameter. For each full-sib population, we delimited a symmetrical interval of ± 3 µm centred around the lowest mode of the pollen diameter across the entire offspring. Pollen falling within this interval was classified as reduced, while larger pollen was classified as unreduced. Smaller pollen was considered malformed and removed from the dataset. We then used this pollen classification to calculate the proportion of unreduced pollen for each observation. To ensure the reliability of this proportion, we excluded samples containing less than 50 pollen grains. We also excluded the observations of two triploid individuals identified in population FRW19-112 and one identified in population IVP16-560. Furthermore, we removed observations from 48 previously characterized desynaptic individuals from population CE-XW (Clot et al. 2023a). After these exclusions, we obtained a total of 81 phenotyped FRW12-112 individuals, 187 phenotyped IVP16-560 individuals (119 in 2019 and 175 in 2020), and 247 phenotyped CE-XW individuals (165 in 2020 and 218 in 2022).

### BLUPs and heritability estimation

We used Generalized Linear Mixed Models (GLMM), allowing for a binomial distribution and random effects, to analyse the level of unreduced pollen production in all three populations. This choice was motivated by the fact that our discrete proportion data violated the linear model assumptions of normality and homoscedasticity of residuals. By allowing a larger variance for intermediate probabilities, the binomial distribution offered a better fit. Therefore, we fitted the model described by Eq. (1) to population IVP16-560 and CE-XW and the simplified model described by Eq. (2) to population FRW19-112. In both models, logit is the link function between the linear predictors ${\eta }_{ijk}$ (or ${\eta }_{ik}$) and the observations ${\pi }_{ijk}$ (or ${\pi }_{ik}$) of the *k*th record of the *i*th genotype in the *j*th year, $\mu$ is the population mean, ${(G}_{i})$ is the random effect of genotype *i*, ${Y}_{j}$ is the fixed effect of year *j*, and $\left(G{Y}_{ij}\right)$ is the genotype *i* by year *j* interaction. The choice of a logit link was motivated by the fact that we could model the probability of a pollen grain to be reduced or unreduced based on the respective count of reduced and unreduced pollen grain in each record. Models were fitted with the R package glmmTMB version 1.1.2.3 (Brooks et al. 2017). We used likelihood ratio tests to assess the significance of random effects via the Chi-square statistic. We tested the significance of the fixed effect of year with the Wald’s test and the t-as-z-approach implemented in glmmTMB. Finally, we extracted predictions for the random genotype effects (BLUPs) for single year and multiyear models and used them as phenotypes in the subsequent QTL analyses.

1 $${\text{logit}}\left( {{\uppi }_{ijk} } \right) = {\upeta }_{ijk} = {\upmu } + (G_{i} ) + Y_{j} + \left( {GY_{ij} } \right)$$

2 $${\text{logit}}\left( {\pi_{ik} } \right) = \eta_{ik} = \mu + \left( {G_{i} } \right)$$

We estimated broad-sense heritability using Eq. (3) (Holland et al. 2002; Piepho and Möhring 2007). where ${\sigma }_{g}^{2}$ is the genotypic variance estimated using the GLMM equations described above and ${\overline{v}}_{\Delta \cdot \cdot }^{{\text{BLUE}}}$ is the mean variance of a difference of two genotypic BLUEs estimated using the same equations but considering ${G}_{i}$ as the fixed effect of genotype *i*.

3 $${\text{H}}^{2} = \frac{{{\upsigma }_{{\text{g}}}^{2} }}{{{\upsigma }_{{\text{g}}}^{2} + {\overline{\text{v}}}_{\Delta \cdot \cdot }^{{{\text{BLUE}}}} /2}}$$

### Whole genome sequencing of parental clones

In a previous study (Clot et al. 2023b), we sequenced clone C and E with paired-end reads, quality trimmed those reads with fastp version 0.19.5 (Chen et al. 2018), aligned them to DM v6.1 with BWA-MEM v.0.7.17 (Li and Durbin 2009), and called variants with bcftools v.1.13 (Danecek et al. 2021). Here, we sequenced the parental clones of population IVP16-560 with 150 bp paired-end reads using BGISEQ-500 and retrieved sequencing reads of RH89-039-16 and IVP10-281-1 from BioProject ID PRJEB2504 and PRJEB36551, respectively. Following the alignment pipeline of Clot et al. (2023b), we obtained a mean haploid coverage of 10.5×, 16.9×, 24.7 ×, and 23.9 × for clone RH89-039-16, IVP10-281-1, IVP92-057-3, and IVP92-030-14, respectively. We then visualised those alignments in candidate gene regions using IGV (Robinson et al. 2011). Finally, we extracted variants identified in the vicinity of *Soltu.DM.07G014730* (± 500 bp) and annotated them using snpEff v4.3 (Cingolani et al. 2012).

### Genotyping by amplicon sequencing and linkage map construction

We generated marker data for all three populations using the amplicon sequencing strategy potatoMASH (Leyva-Pérez et al. 2022). DNA extraction, library preparation, and bioinformatic processing of sequencing reads to obtain marker dosages were performed according to Leyva-Pérez et al. (2022), with the exception that we aligned short-reads to DM v6.1 (Pham et al. 2020) instead of DM v4.03. Unique amplicon alleles, referred to as haplotags, were used as genetic markers. We obtained a total of 890, 822, and 873 segregating markers for population FRW19-112, IVP16-560, and CE-XW, respectively. Next, we used polymapR version 1.1.2 (Bourke et al. 2018) and followed the package vignette to automate the linkage map construction in all three populations. First, we removed nonsegregating markers and markers with high missing rates (> 20%) or strongly distorted transmission ratios. When possible, we manually imputed missing parental dosages based on offspring segregation. We also removed poorly genotyped individuals missing more than 20% of markers. Next, we calculated recombination frequencies for all marker-pairs, clustered the markers into 12 chromosomal groups, and ordered them via multidimensional scaling (Preedy and Hackett 2016). Finally, we phased the markers into parental homologues using PolyoriginR version 0.0.3 (Zheng et al. 2021). The final phased linkage maps were composed of 755, 655, and 737 markers segregating across 91, 186, and 233 individuals for populations FRW19-112, IVP16-560, and CE-XW, respectively (Table S1). Using Marey maps (Chakravarti 1991) (Figure S2-4), we confirmed that the genetic order of our linkage maps follows the expected physical order of potatoMASH amplicons on DM v6.1 except for a previously described paracentric inversion on the long arm of chromosome *3* (Tang et al. 2022).

### QTL mapping

We combined BLUPs and phased linkage maps in polyqtlR version 0.0.9 (Bourke et al. 2021) to perform a QTL analysis following the package vignette. We estimated offspring Identity-by-descent (IBD) probabilities using a Hidden Markov Model with a prior error ranging from 0.01 to 0.05 per chromosome. We then performed an IBD-based QTL analysis with the QTLscan function by regressing genotypic BLUPs on the parental homologue probabilities. LOD significance thresholds were determined via permutation tests on the BLUPs values with *N* = 1000 cycles and *α* = 0.05. Next, we identified markers significantly associated with unreduced pollen production and examined the effect of marker dosage on unreduced pollen production using the nonparametric Kruskal–Wallis test and the Conover-Inman post hoc test (*α* = 0.05) corrected for multiple comparison with Holm procedure.

### Candidate genes exploration

We delimitated a $LOD-1.5$ confidence interval around each QTL peak and extracted the physical position of markers flanking this interval. When those flanking markers corresponded to the first or last marker of the linkage group, the physical confidence interval was extended to the most distal coordinates of the chromosome. We then narrowed the candidate region for each major-effect QTL by intersecting confidence intervals across years and populations. Next, we combined the high confidence gene model annotation of DM v6.1 with their GOSlim annotations and descriptions based on their best hit with the *A. thaliana* proteome TAIR10. We then retrieved all DM v6.1 annotated genes within our candidate regions and filtered them by GOSlim terms for reproduction (GO:0000003) or cell cycle (GO:0007049). Finally, we manually reviewed the TAIR10 curator summary and computational description of those short-listed genes to identify candidate genes for unreduced pollen production.

### Sequence variation in genes encoding for JASON-RELATED proteins

To retrieve *StJR1* alleles from DM v6.1 and Otava (Sun et al. 2022), we constructed a local BLAST database with these two assemblies using Nucleotide-Nucleotide BLAST 2.8.1 + (Camacho et al. 2009). We queried against this database the genomic sequence of *Solyc07g045010.3.1*, the *JR1* allele of *S. lycopersicum* cv. Heinz 1706 SL4.0 genome (Su et al. 2021), with default parameters. We manually curated the results by stitching together partial hits within 5-kb of each other and extracted genic and 500-bp upstream and downstream regions homologous to *Solyc07g045010.3.1*. We performed a multiple sequence alignment of *StJR1* alleles and *Solyc07g045010.3.1* coding sequence in Geneious Prime 2022.2.2 (https://www.geneious.com) using MUSCLE 3.8.425 (Edgar 2004). Based on this alignment we translated *JR1* exons using the build-in translate function of Geneious Prime 2022.2.2. To investigate allelic variation in *StJR2*, we blasted on EnsemblPlants (Yates et al. 2022) with default parameters the amplicon allele associated with high level of unreduced pollen on chromosome *12* against RH89-039-16 v3 (Zhou et al. 2020). We then retrieved the protein sequence of the *StJR2* alleles in coupling and repulsion phase with this amplicon. We aligned those protein sequences, together with a set of JASON-RELATED proteins from the panel of plants used by Erilova et al. (2009) using MUSCLE 3.8.425.

### Language editing

ChatGPT (GPT-3, OpenAI’s large-scale language-generation model) was used for language editing. ChatGPT edits were reviewed and revised by the authors who take ultimate responsibility for the content of this publication.

## Results

### Misorientation of metaphase II plate observed in parental clones

Mok and Peloquin (1975a) and Ramanna (1979) studied the meiosis of the historical clones C (USW5337.3) and E (77.2102.37), the parents of our CE-XW population. Both studies report the presence of parallel and fused metaphase II (MII) spindles and, more generally, the misorientation of MII plate causing the formation of unreduced pollen. It is also known that the unreduced pollen produced by RH89-039-16 is genetically equivalent to FDR (Park et al. 2007), a typical outcome of MII plate misorientation. Although we suspected that MII plate misorientation was also the cause of unreduced pollen production in clones RH89-039-16 and IVP92-057-3 due to their shared ancestry with clones C and E, we opted to validate this assumption through cytological observations. We used the chromosome spread technique to examine the meiotic progression and chromosomal behaviour of clones RH89-039-16 and IVP92-057-3 known to produce unreduced pollen, and of clones IVP10-281-1 and IVP92-030-14 that are not able to do so. As expected, clones RH89-039-16 and IVP92-057-3 produced a mixture of tetrads (50% and 30%), dyads (45% and 62%), and triads (3% and 7%), and clones IVP10-281-1 and IVP92-030-14 essentially produced only tetrads (98% and 99%) (Figure S5). For clones RH89-039-16 and IVP92-057-3, we also occasionally observed the presence of nuclear fusion during telophase II resulting in 2% and 1% of unbalanced products (Figure S5). The meiotic progression of clone IVP92-057-3 was asynchronous, with early meiotic stages such as pachytene and diakinesis occasionally observed next to dyads, which could suggest occasional omission of the second meiotic division. This was not the case for the three other clones where only consecutive meiotic stages could be observed simultaneously (Figure S5).

**Fig. 1:**
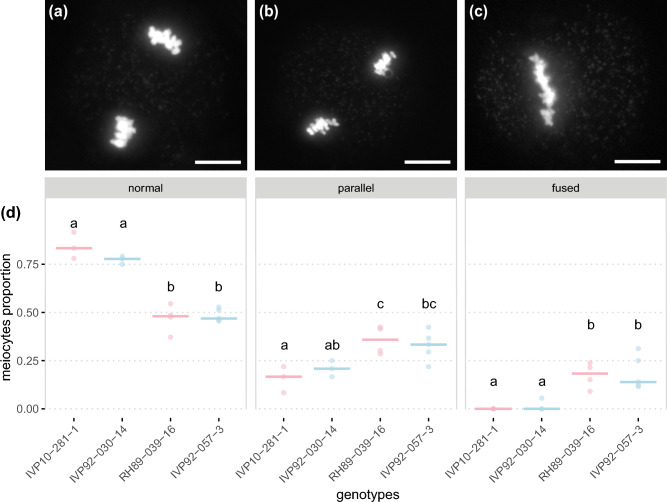
Distinctive metaphase II orientation phenotypes in parental clones. **a**–**c** DAPI-stained chromosome spreads of male meiocytes at metaphase II. **a** Normal and **b** parallel orientation of metaphase II plates, **c** fused metaphase II plates. (d) Proportion of meiocytes with normally oriented (left), parallel (middle), or fused metaphase II plates (right) for the parental clones of population FRW19-112 in pink and IVP16-560 in blue. Median values per orientation and per clone are indicated by vertical bars. Significantly different median values between clones for a given orientation are indicated by different letters. Scale bars = 10 µm

After this general outlook on the progression of meiosis, we focused our attention on MII. During this stage, the chromosomal behaviour of the two clones producing unreduced pollen differs from the other two clones. Roughly 80% of the meiocytes of clones IVP10-281-1 and IVP92-030-14 showed the expected perpendicular orientation of MII plates, while the remaining ~ 20% displayed a parallel orientation. In contrast, for the unreduced pollen producing clones RH89-039-16 and IVP92-057-3, only ~ 50% of meiocytes had normally oriented MII plates, with the other half of the meiocytes displayed either parallel or fused MII plates. Approximately 35% of their meiocytes exhibited parallel MII plates, which correspond to a 1.75 fold increase when compared with the ~ 20% reported for IVP10-281-1 and IVP92-030-14. We observed an even more striking increase in the proportion of meiocytes with fused MII plates, which represented ~ 15% of RH89-039-16 and IVP92-057-3 meiocytes, while this phenotype was virtually absent in clones IVP10-281-1 and IVP92-030-14 (Fig. 1). Overall, misorientation of MII plates appears to be the main meiotic restitution mechanism of RH89-039-16 and IVP92-057-3. Nonetheless, the presence of additional restitution mechanisms such as partial omission of meiosis II and fusion of nuclei cannot be excluded.

### Proportion of unreduced pollen production is a heritable quantitative trait

**Fig. 2:**
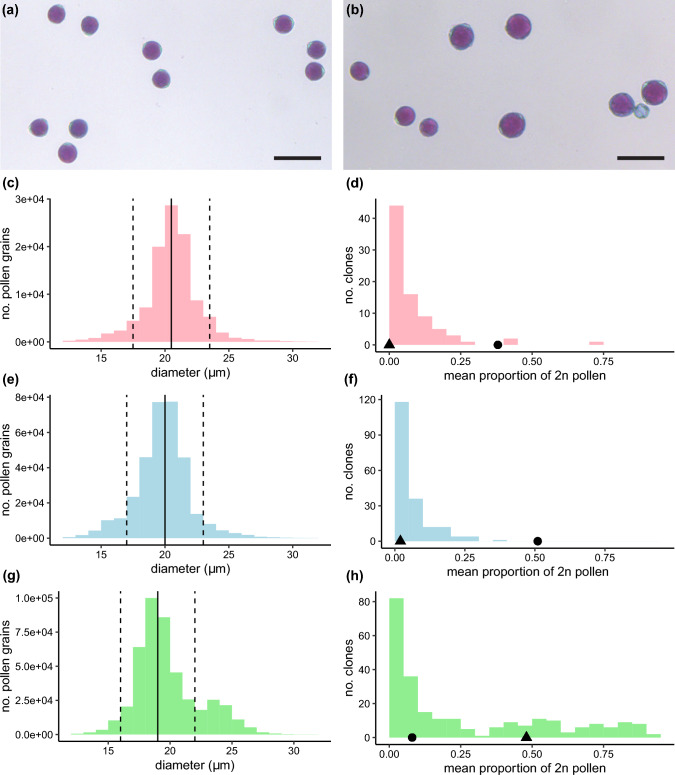
Microscope pictures of (**a**) normal reduced pollen, and (**b**) a mixture of reduce and unreduced pollen. The scale bar represents 50 µm. Distribution of pollen diameter (**c**, **e**, **g**) and proportion of unreduced pollen (**d**, **f**, **h**) are shown in pink for population FRW19-112, in blue for IVP16-560, and in green for CE-XW. The vertical dashed lined represents the 3-µm symmetrical interval centred around the mode of reduce pollen diameter indicated by the full vertical line. For each population, maternal and paternal mean unreduced pollen proportion are indicated by a circle and a triangle, respectively. Scale bars = 50 µm

Such detailed cytological observations are not scalable to entire populations. Therefore, we exploited the correlation between pollen size and ploidy level to phenotype unreduced pollen production using microscopic image analysis of pollen grains (Fig. 2a-b). In the CE-XW population, the distribution of pollen diameter was bimodal (Fig. 2g), with the first mode corresponding to reduced haploid pollen and the second mode to unreduced diploid pollen. However, in populations FW19-112 and IVP16-560, the relatively lower proportion of unreduced pollen did not result in a visible second mode (Fig. 2c, e). The mode of reduced pollen diameter showed mild variation across populations, with values approximately centred around 19 μm, 20 μm, and 20.5 μm for CE-XW, IVP16-560, and FRW19-112, respectively. Taking this into consideration, we defined a symmetrical interval of ± 3 µm around the mode of reduced pollen diameter in each population. Pollen falling within this interval was classified as reduced, while larger pollen was classified as unreduced. Next, we calculated the proportion of unreduced pollen for each observation and plotted the distribution of mean unreduced pollen production per clone for the three populations (Fig. 1d,f,h). Interestingly, all three distributions were skewed toward the left with 54%, 63%, and 33% of the offspring in populations FRW19-112, IVP16-560, and CE-XW producing less than 5% unreduced pollen. The highest levels of unreduced pollen production were observed in population CE-XW, with 26% of clones producing over 50% unreduced pollen. Such levels of unreduced pollen were virtually absent in the other two populations. Overall, the distribution of the proportion of unreduced pollen produced was continuous in the three populations. As it was challenging to define a threshold to classify descendants with or without unreduced pollen, we chose to treat the proportion of unreduced pollen as a quantitative trait.

As expected with discrete proportions, our data violated the linear model assumptions of normality and homoscedasticity of residuals. Indeed, the variance between repeated observations in time and across growing seasons was higher for clones producing over 10% of unreduced pollen than for those producing less (Figure S6-S8). We therefore used GLMM Eq. (1) (which is simplified to Eq. (2) for FRW19-112) with a logit link and a binomial distribution to fit our data. In all three populations, the variable genotype explained most of the variance. Inclusion of the genotype by year interaction improved model accuracy for populations IVP16-560 and CE-XW (Table 1). The fixed effect of year was also significant for those two populations, particularly for population IVP16-560. Consistent with the visual examination of the data (Figure S7), population IVP16-560 showed a higher intercept for the field trial of 2020 compared with the greenhouse one of 2019. We then extracted genotypic BLUPs for subsequent QTL analysis and estimated broad-sense heritability using the genetic variance (${\upsigma }_{{\text{g}}}^{2}$) and the mean variance of difference between BLUEs (${\overline{{\text{v}}}}_{\Delta \cdot \cdot }^{{\text{BLUE}}}$) as part of Eq. (3). We treated genotypes as random effect while extracting genotypic BLUPs and estimating ${\upsigma }_{{\text{g}}}^{2}$. On the other hand, we treated them as fixed effect while estimating ${\overline{{\text{v}}}}_{\Delta \cdot \cdot }^{{\text{BLUE}}}$, which measures the precision of pairwise comparisons. We obtained a high broad-sense heritability in all three populations with estimates of 0.971 for FRW19-112, 0.896 for IVP16-560, and 0.923 for CE-XW, indicating a strong influence of the genotype on the level of unreduced pollen production.

**Table 1:** Partitioning of variance components and broad-sense heritability estimates for unreduced pollen production in the three mapping populations

	FRW19-112		IVP16-560		CE-XW	
**Random Effect**	**Var. Comp.**	**Std Dev**	**Var. Comp.**	**Std Dev**	**Var. Comp.**	**Std Dev**
Genotypes	2.672	1.635	1.236**	1.112	4.239**	2.059
genotypes*year	NA	NA	0.429**	0.655	1.293**	1.137
**Fixed Effect**	**Estimate**	**Std Error**	**Estimate**	**Std Error**	**Estimate**	**Std Error**
Intercept	− 3.341	0.184	− 4.023	0.111	− 1.816	0.166
2nd year	NA	NA	0.818**	0.094	− 0.229*	0.136
${\overline{{\text{v}}}}_{\Delta \cdot \cdot }^{{\text{BLUE}}}$	0.158		0.286		0.714	
${H}^{2}$	0.971		0.896		0.923	

^**^ Significant with p-value < 0.001; * Significant with p-value < 0.05

### Several QTLs regulate unreduced pollen production, two co-localise with AtJAS homologs

We used an amplicon sequencing approach to genotype our three populations and generate phased linkage maps (Table S1). We then identified QTLs for unreduced pollen production by regressing genotypic BLUPs against parental homolog probabilities. Seven QTLs were identified: one in population FRW19-112, four in population IVP16-560, and two in population CE-XW (Table 2, Figure S9). Notably, we detected a large-effect QTL on the long arm of chromosome *12* in all three populations (Fig. 2a), which explained 25.1%, 26.2%, and 29.0% of BLUPs variance in FRW19-112, IVP16-560, and CE-XW, respectively. Additionally, we identified two population-specific large-effect QTLs: *q2nP_IVP_5* (for QTL 2n pollen IVP16-560 chromosome *5*) and *q2nP_CE_7* (for QTL 2n pollen CE-XW chromosome 7), which explained 19.0% and 38.9% of BLUPs variance, respectively. All those QTLs were consistently detected across years in populations IVP16-560 and CE-XW. In population IVP16-560, we also found two lesser-effect QTLs on chromosome *9* and *11*, each explaining less than 10% of multiyear BLUPs variance.

**Table 2:** Summary of QTLs for unreduced pollen production identified in the three mapping populations

QTL ID^1^	Population	Size^2^	Year	LOD	Chr	Pos^3^ (cM)	PVE^4^	CI^5^ (Mbp)
*q2nP_FRW_12*	FRW19-112	78	2020	4.9	12	61.4	25.1%	51.4–59.5
*q2nP_IVP_19_5*	IVP16-560	113	2019	6.9	5	58.2	24.6%	51.1–55.5
*q2nP_IVP_19_11*	IVP16-560	113	2019	3.5	11	12.3	13.4%	0–41.3
*q2nP_IVP_19_12*	IVP16-560	113	2019	6.2	12	44.0	22.2%	10.2–55.5
*q2nP_IVP_20_5*	IVP16-560	170	2020	7.5	5	58.2	18.3%	50.2–55.5
*q2nP_IVP_20_9*	IVP16-560	170	2020	4.7	9	6.8	11.9%	0–3.7
*q2nP_IVP_20_11*	IVP16-560	170	2020	4.8	11	40.1	12.3%	10.8–43.1
*q2nP_IVP_20_12*	IVP16-560	170	2020	5.5	12	42.7	13.9%	50.2–54.7
*q2nP_IVP_5*	IVP16-560	180	combined	7.5	5	58.2	19.0%	51.1–55.5
*q2nP_IVP_9*	IVP16-560	180	combined	4.7	9	6.8	9.0%	0–64.6
*q2nP_IVP_11*	IVP16-560	180	combined	4.8	11	40.1	9.5%	10.0–46.8
*q2nP_IVP_12*	IVP16-560	180	combined	5.5	12	42.7	16.2%	10.2–55.5
*q2nP_CE_20_7*	CE-XW	117	2020	10.7	7	42.6	34.2%	43.4–46.2
*q2nP_CE_20_12*	CE-XW	117	2020	5.6	12	61.1	19.9%	52.8–58.5
*q2nP_CE_22_7*	CE-XW	171	2022	17.2	7	43.6	37.1%	43.4–46.2
*q2nP_CE_22_12*	CE-XW	171	2022	13.0	12	61.1	29.6%	52.8–56.2
*q2nP_CE_7*	CE-XW	187	combined	19.6	7	42.6	38.4%	43.4–46.2
*q2nP_CE_12*	CE-XW	187	combined	13.9	12	61.1	29.0%	52.8–55.5

^1^QTL the format “q2nP_pop_year_chrom” describing the phenotype, the segregating_population, the_year (optional) and_chromsome, where year is omitted when phenotypes across all years are combined to predict genotypic BLUPs ^2^Size refers to the effective population size tested across years; ^3^Pos refers to the genetic position of QTLs; ^5^PVE refers to the percentage of variance explained by the QTLs; ^5^CI refers to the $LOD-1.5$ physical confidence interval around QTLs

Our amplicon sequencing method results in marker loci with multiple alleles referred to as haplotags. In all three populations, the haplotag representing the sequence of the DM reference genome, located at ~ 53.8 Mb on DM v6.1 chromosome *12*, was associated with a high proportion of unreduced gametes. Individuals homozygous for this DM allele had the highest level of unreduced pollen with a median value of 13.0% for FRW19-112, 42.2% for CE-XW, and 7.5% for IVP16-560 (Fig. 2b-d). In the latter population, individuals heterozygous or without the DM allele showed a similarly low proportion of unreduced pollen with median values of 2.7% and 2.4%, respectively, suggesting a recessive mode of inheritance. However, this could not be confirmed in population FRW19-112, where the inheritance appears to fit a dominant model despite the median being 2.5-fold higher for DM homozygotes than for heterozygotes. This discrepancy could be due to the smaller population size of FRW19-112 (*n* = 77) and the transmission ratio distortion observed against the DM allele (Fig. 2b). We observed that FRW19-112 and IVP16-560 parents are heterozygous for the DM allele and therefore that *q2nP_FRW_12* and *q2nP_IVP_12* segregate from both parents. In contrast, in population CE-XW, *q2nP_CE_12* segregated from the parental clone E only, while the parental clone C is homozygous for the DM allele. This suggests that *q2nP_CE_12* is contributing to the formation of unreduced pollen in clone C.

For *q2nP_CE_7,* the haplotag located at ~ 44.3 Mb on chromosome *7* and associated with elevated unreduced pollen production was also identical to DM*.* We observed a tenfold increase in unreduced pollen production between individuals homozygous and heterozygous for the DM haplotag, indicating compatibility with a recessive model of inheritance (Fig. 2f). This QTL segregated from clone C only, while clone E was homozygous for the DM allele, suggesting that *q2nP_CE_7* is contributing to unreduced pollen production in clone E. In contrast, *q2nP_IVP_5* segregated from both parents and appears to follow an additive model (Fig. 2e).

**Fig. 3:**
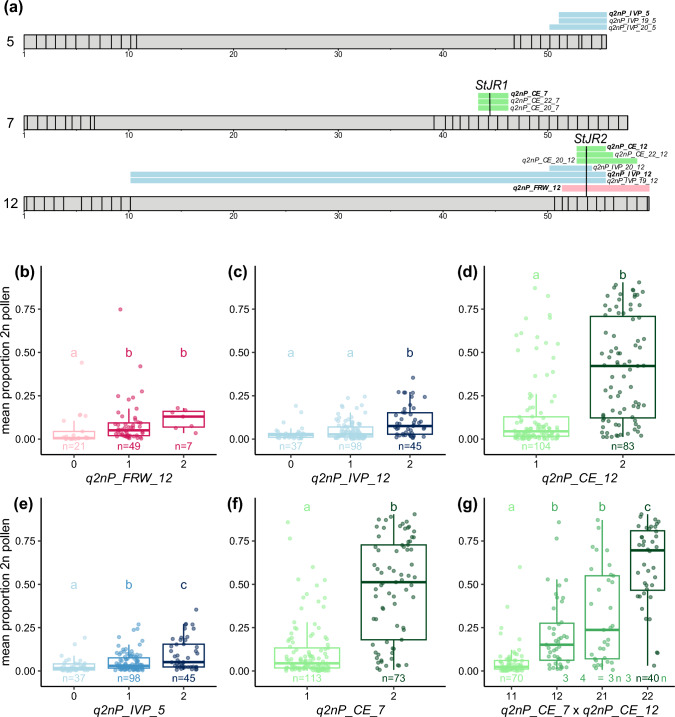
Position and effect of three major effect QTL involved in unreduced pollen production. **a** Horizontal bars present the physical length of potato chromosomes (in Mb) including the marker positions in euchromatic chromosome arms as vertical lines. The $LOD-1.5$ confidence intervals around major-effect QTLs in population FRW19-112, IVP16-560, and CE-XW are shown in pink, blue, and green, respectively. QTLs detected based on all years data are indicated in bold. **b**–**g** Dosage effect of haplotags associated with unreduced pollen production for each major-effect QTL. Each point represents the mean level of unreduced pollen production of one clone. Significantly different median trait values between haplotag dosage groups (*α* = 0.05) are indicated with different letters and colour shades (color figure online)

Finally, we analysed dosage variation at *q2nP_CE_7* and *q2nP_CE_12* simultaneously (Fig. 2g). Individuals heterozygous for the DM allele at both loci produced the lowest proportion of unreduced pollen (median of 2.4%), while individuals homozygous for the DM haplotag at either locus produced a significantly higher proportion of unreduced pollen (median of 23.7% for *q2nP_CE_7* and 15.2% for *q2nP_CE_12*). Hence, taken independently, the effect of *q2nP_CE_12* was of the same order of magnitude as *q2nP_FRW_*12 and *q2nP_IVP_*12. Remarkably, individuals homozygous for both QTLs produced a median level of unreduced pollen of 69.6%, a value 29-fold higher than that of double heterozygotes, suggesting at least additivity and potentially synergistic interaction between *q2nP_CE_7* and *q2nP_CE_12* (Figure 3).

To identify candidate genes associated with unreduced pollen production, we used a $LOD-1.5$ confidence interval around the major effect QTLs (Table 2) and narrowed three candidate gene regions by intersecting overlapping intervals. This resulted in a 4.5 Mb region on chromosome *5* (from ~ 51.1 to ~ 55.6 Mb), a 2.8 Mb region on chromosome *7* (from ~ 43.4 to ~ 46.2 Mb), and a 1.9 Mb region on chromosome *12* (from ~ 52.8 to ~ 54.7 Mb). We then extracted DM v6.1 annotated genes in these regions and selected the ones predicted to be involved in cell cycle or reproduction based on homology with *A. thaliana* genes (Table S2). We identified 454 genes within the candidate region on chromosome *5*, including 36 involved in cell cycle or reproduction. Among them, we found no genes with a direct link to unreduced gametes. However, we identified three genes worth noting: *Soltu.DM.05G022860* with homology to *AtPOR* involved in tubulin complex assembly and cytokinesis (Mayer et al. 1999), *Soltu.DM.05G023410* with homology to *AtRGL2* involved in gibberellic acid signalling, and *Soltu.DM.05G023970* with homology to *AtRAD50* involved in meiotic double-strand break repair (Gallego et al. 2001). The candidate region on chromosome *7* contained 166 genes including 13 involved in cell cycle or reproduction. Among them we identified *Soltu.DM.07G014730* as the most likely candidate gene due to its homology with the meiotic regulator *AtJAS* whose mutants are known to produce unreduced pollen by restitution of the first division (Erilova et al. 2009; De Storme and Geelen 2011). Finally, in the candidate region on chromosome *12* we found 167 genes including 16 involved in cell cycle or reproduction. Strikingly, another gene, *Soltu.DM.12G023840,* with homology *AtJAS* was found. Following the naming convention of Cabout et al. (2017), we will refer to *Soltu.DM.07G014730* and *Soltu.DM.12G023840* as alleles of *StJR1* and *StJR2* (for *S. tuberosum JASON-RELATED 1* and *2*), respectively.

### Sequence variation across StJR1 and StJR2 alleles suggests functional defects

**Fig. 4:**
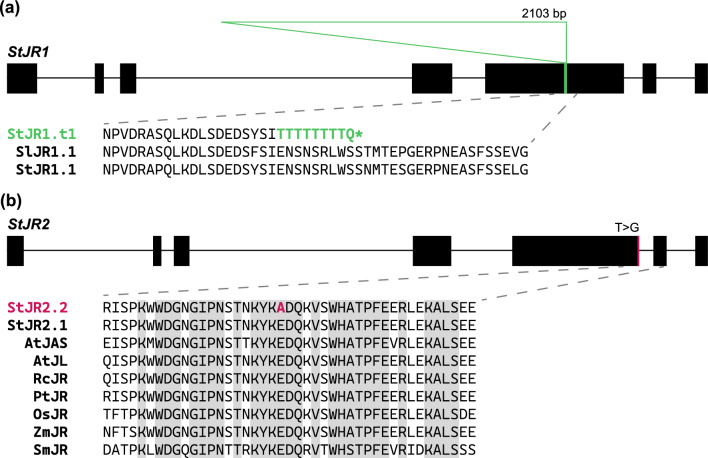
Functional and mutant *StJR1* and *StJR1alleles* (**a**) diagram of the exon intron structure of *StJR1*. The position of the deletion identified in *StJR1.t1* is highlighted by a green stripe and the position of the insertion by a green triangle. The sequence of JR proteins overlapping with the indel site is displayed for the DM allele (*StJR1.t1*), the tomato *Solyc07g045010.3.1* allele (*SlJR1.1*) and the Otava *St2-St07G406640* allele (*StJR1.1*). **b** Diagram of the exon intron structure of *StJR2*. The position of the missense mutation associated with high levels of unreduced gamete production in RH89-039-16 is highlighted by a pink stripe. The C-terminal sequence of a set of JASON-RELATED proteins overlapping with the missense mutation is displayed. Amino acid residues with 100% sequence conservation are highlighted in grey. At: *Arabidopsis thaliana*, Rc: *Ricinus communis*, Pt: *Populus trichocarpa*, Os: Oryza sativa, Zm: *Zea mays*, Sm: *Selaginella moellendorffii* (color figure online)

Sequence variation in the candidate genes *StJR1* and *StJR2* was examined to identify variants with putative effect on the functionality of the JASON-RELATED proteins. Upon closer inspection of the annotation of the reference genome DM v6.1 in the *StJR1* region, we noticed that two neighbouring genes separated by 2109 bp, *Soltu.DM.07G014730* and *Soltu.DM.07G014740*, were annotated as *AtJAS* homologs. The structure of those two genes, each with five exons, differed from the *JR1* gene structure having seven exons in *A. thaliana*, *S. lycopersicum* and in other potato clones such as Otava. Once *Soltu.DM.07G014730* and *Soltu.DM.07G014740* were aligned with the corresponding genomic regions in Otava and with the coding sequence of the tomato *JR1* gene *Solyc07g045010.3.1* we realized that the annotation in DM v6.1 was affected by a 16-bp deletion and a 2103-bp insertion in the 5th exon of *StJR1* resulting in two partial *JR1* genes. We queried this insertion against the RepetDB database (Amselem et al. 2019) which classified it as a Class I LINE transposon. Hence, we will from now on refer to the DM allele of *StJR1 as StJR1.t1* where t stands for transposon. In silico translation of the disrupted 5th exon of *StJR1.t1* led to a premature stop codon (Fig. 4a). The visual examination of clones C and E short reads aligned to DM v6.1 confirmed that clone E is homozygous for the *StJR1.t1* allele, while clone C is heterozygous (Figure S10). Next, we retrieved from the genome assembly of RH89-039–16 (the maternal clone of population FRW19-112) *StJR2* alleles in coupling and repulsion phase with the chromosome *12* haplotag associated with high levels of unreduced pollen. Assuming that *StJR2* is the ca

usal gene behind *q2nP_FRW19-112_12*, the allele in repulsion phase with this haplotag (*RHC12H2G0793.2*, from now on *StJR2.1*) must be wild type, while the allele in coupling phase (*RHC12H1G0690.2*, from now on *StJR2.2*) must be mutant. We noted 18 amino acid changes between the StJR2.1 and StJR2.2 proteins of which one, the conversion of glutamine to an alanine (Glu > Ala) at position 432, was located within the highly conserved C-terminal domain of JASON-RELATED proteins (Fig. 4b) (Erilova et al. 2009). This Glu > Ala conversion is due to a T > G missense mutation on the second last nucleotide of *StJR2* 5th exon, located at 53,693,790 bp on DM v6.1, and annotated by snpEff as having a moderate effect on StJR2. Changing a polar glutamine by a nonpolar alanine in such a conserved region is likely to affect the function of this protein. Consistently with the identification of QTLs for unreduced pollen production in this region of chromosome *12* in our three mapping populations, we found back the T > G SNP in a heterozygous condition in the six parental clones of our three mapping populations (Figure S11). While this explains the bi-parental segregation observed for *q2nP_FRW_12* and *q2nP_IVP_12*, it is insufficient to account for the uniparental segregation observed for *q2nP_CE_12*.

## Discussion

### Pollen diameter as a proxy for pollen ploidy

Our phenotyping approach, based on pollen microscopy, heavily relies on the premise that pollen size correlates with pollen ploidy. This correlation has been consistently reported across various species (Ramsey and Schemske 1998), including in potato (Mok and Peloquin 1975a, b; Ramanna 1979; Iwanaga and Peloquin 1982; Dongyu et al. 1995) where it is routinely used by breeders for designing interploidy crosses (Zimnoch-Guzowska and Flis 2021). However, it is essential to acknowledge that factors beyond ploidy may influence pollen size. These factors could be either of genetic origin, as suggested by a mean haploid pollen diameter variation of up to 1.5 μm across our mapping population (Fig. 2), or environmental, such as pollen grain desiccation. Despite these considerations, we maintain confidence in identifying large pollen grains as unreduced (2n) for several reasons. Firstly, the parental clones producing large pollen grains also exhibit an elevated proportion of dyads (Figure S5). Secondly, in CE-XW clones lacking crossovers, the presence of large pollen grains effectively restored fertility, aligning with the expected outcome of a restitution of the first meiotic division (Clot et al. 2023a). Lastly, we identified candidate genes directly linked with meiotic restitution for two of the three major QTLs uncovered in this study.

### The intricate layers of complexity of unreduced pollen production in potato

Production of unreduced gametes is undeniably a complex trait, influenced both by environmental and genetic factors (De Storme and Geelen 2013, 2014). In this study, the main cause of variation was genetic as indicated by the high broad sense heritabilities observed in our three-mapping populations. However, even when environmental influence is accounted for, the genetic basis of unreduced pollen in potato remains complex. The first layer of complexity is a mechanistic one. Unreduced pollen can result from various meiotic restitution mechanisms. While in mutagenized *A. thaliana* such mutants can be studied independently or in controlled combinations, in potato clones several meiotic alterations can co-occur (Ramanna 1979). We aimed to reduce this variability by working with parental clones sharing ancestry with the historical clones C and E, known to produce unreduced pollen via misorientation of MII plates and spindles (Mok and Peloquin 1975b; Ramanna 1979). With cytological observations we confirmed that clone RH89-039-16 and IVP92-057-3 indeed displayed this restitution mechanism. However, we also observed the co-occurrence of additional meiotic aberrations such as partial omission of the second division and nuclear fusion. The second layer of complexity is linked with the quantitative phenotype of unreduced pollen production. In all three populations, variation in unreduced pollen production was continuous. Therefore, simple classification into producers and nonproducers of unreduced pollen depends on an arbitrarily threshold. This has happened in earlier work, notably with inconsistent threshold values across studies (Mok and Peloquin 1975a, b; Iwanaga and Peloquin 1982). This threshold-based classification endorsed the conclusion that unreduced pollen production in potato was under the control of independent monogenic and recessive mutations, one affecting spindle orientation and two affecting cytokinesis (Mok and Peloquin 1975a). In contrast, we adopted a quantitative approach, as previously advocated by Dongyu et al. (1995), and identified two minor-effect and three major-effect QTLs involve in unreduced pollen production. We also expect additional QTLs underlying MII plates and spindle orientation, but not segregating in our populations. Indeed, clones RH89-039-16 and IVP92-057-3 were both heterozygous at all QTLs positions while producing about 38% and 51% unreduced pollen, respectively. Overall, the involvement of at least 5 loci in unreduced pollen production follows the multiple mechanisms of meiotic restitution and the involvement of several genes for each mechanism. The third layer of complexity is allelic heterogeneity. For any given QTL and the underlying candidate gene, more than one haplotype can be mutant, based on different sequence variants. This could be the case for *q2n_CE_12,* for which both alleles of clone C contribute to unreduced pollen production, while this clone is heterozygous for the T > G missense mutation in *StJR2.2*, suggesting the presence of a second nonfunctional allele. Taken together, these layers of complexity explain why, prior to this study, the genetic regulation of unreduced pollen in potato has remained elusive for more than 50 years after the descriptions of an allegedly monogenic Mendelian inheritance.

### AtJAS homologs as candidate genes

The misorientation of MII plates and spindles is the most frequently reported mechanism of male meiotic restitution in plants (Bretagnolle and Thompson 1995; De Storme and Geelen 2013). Nonetheless, only three genes, *AtPS1* (D’Erfurth et al. 2008), *AtJAS* (De Storme and Geelen 2011) and *AFH14* (Li et al. 2010), are known to be involved MII spindle and plate orientation. While *AFH14* regulates microtubule and microfilament arrays, *AtJAS* and *AtPS1* form a mini regulatory network controlling MII spindle orientation. Although similar in gametic outcome, the loss-of-function phenotype of *Atjas1* differs from *Atps1* in the unique loss of the MII organelle band required to prevent spindle interaction (Brownfield et al. 2015). In *A. thaliana* two forms of AtJAS are produced: a long version with an N-terminal Golgi localization signal and a short version localizing to the plasma membrane (Cabout et al. 2017). The short version of *AtJAS* is upregulated during meiosis and therefore assumed to be the one responsible for the organelle band maintenance. Another protein, AtJL (for *A. thaliana* JASON-like), presents a high degree of sequence similarity with AtJAS, notably within the conserved C-terminal domain, but lacks the N-terminal Golgi extension (Erilova et al. 2009; Cabout et al. 2017). Although *AtJL* function is unknown, its predominant expression during reproductive development and its localisation to the plasma membrane suggest a meiotic function similar to *AtJAS*.

In this study, we identified the misorientation of MII plates as the principal meiotic restitution mechanism in the parents of our mapping populations. This phenotype fits well the two candidate genes we identified, both showing homology to *AtJAS*: *StJR1* located within the candidate region of *q2nP_CE_7* and *StJR2* located within the shared candidate region of *q2nP_FRW_*12, *q2nP_IVP_*12, and *q2nP_CE_*12. Candidate gene *StJR1* presents the typical N-terminal extension of *AtJAS* and is therefore its putative ortholog*.* Mutagenized *AtJAS* alleles with a premature stop codon prior to the conserved C-terminal domain, in the 3rd or 5th exon, lead to the formation of unreduced pollen in *A. thaliana* (Erilova et al. 2009). Similarly, the transposon insertion in *StJR1.t1*, leading to a premature stop, is located upstream of this conserved C-terminal domain and is likely the causal polymorphism behind *q2nP_CE_7*. On the other hand, candidate gene *StJR2* is lacking the N-terminal Golgi localization signal and is therefore a putative *AtJL* orthologue. *AtJAS*, *AtJL*, and their homologs in other plant species share a highly conserved C-terminal domain (Erilova et al. 2009). Although the function of this C-terminal domain is as yet unknown, its conservation throughout vascular plants suggests importance. In our three mapping populations, a Glu > Ala substitution within this conserved domain was associated with unreduced pollen production, but molecular validation is needed to demonstrate with certainty that this amino acid replacement is the causal mutation behind *q2nP_FRW_*12, *q2nP_IVP_*12, and *q2nP_CE_12*. More generally, this would indicate that JR1 and JR2 proteins are equally important for proper MII plate and spindle orientation. Indeed, in single mutants one functional JR protein seems to partially compensate for loss of the other JR protein, while in double mutants the severity of the phenotype drastically increases.

### Interploidy breeding with unreduced gametes

Unreduced gametes have been successfully used in potato breeding program to introduce genes conferring resistance to various pathogens from diploid wild relatives of potato to tetraploid landraces and cultivars (Ortiz et al. 1994; Capo et al. 2002; Zimnoch-Guzowska and Flis 2021). An interploidy breeding scheme combining the ease of diploid breeding and the heterozygosity of tetraploids was proposed 60 years ago (Chase 1963; Hutten et al. 1994). However, such a breeding scheme has not been adopted widely, partly due to the lack of understanding of the genetic control of unreduced gamete production (Ortiz and Peloquin 1992; Dongyu et al. 1995). The identification of major-effect QTLs and candidate genes underlying unreduced pollen production enables, for the first time, marker-assisted selection (MAS) for this trait. In addition, a dominant gene conferring self-compatibility to diploid potato was identified (Hosaka and Hanneman 1998; Clot et al. 2020; Eggers et al. 2021; Ma et al. 2021), prompting the potato breeding community to move towards a transition from tetraploid clonal selection to a diploid F1 hybrid breeding system (Jansky et al. 2016; Lindhout et al. 2016; Bethke et al. 2022). F1 hybrid breeding is still in its early stages, and large-scale breeding efforts are required to overcome inbreeding depression due to a high genetic load (Zhang et al. 2019, 2021; Comai 2023). In this context, MAS for unreduced pollen production provides the opportunity to cross improved diploids with tetraploid elite material, allowing breeders to select clonally propagated tetraploid varieties, while the diploid F1 hybrid system matures. MAS for unreduced pollen can also be combined with MAS for reduced meiotic recombinations (previously referred to as desynapsis) (Jongedijk and Ramanna 1989; Clot et al. 2023a) to facilitate to the creation of diploid clones producing near nonrecombinant FDR male gametes. These relatively uniform and heterozygous gametes could be used to produce relatively uniform tetraploid varieties grown from true seeds, by crossing a heterozygous diploid clone with a partially inbred tetraploid clone.

Finally, the additivity of *q2nP_CE_7* and *q2nP_CE_*12 offers breeders the ability to fine-tune the level of unreduced pollen production in their clones. The optimal level of unreduced pollen needed to simultaneously allow high seed set at the diploid and tetraploid level is currently unknown. Nevertheless, unpublished data from the crossing booklets of the Wageningen University potato breeding program indicate that for interploidy crosses, as little as 5% of stainable unreduced pollen is sufficient to induce a seed set comparable to a normal tetraploid cross. Similar observations have been made in *Rosa hybrida* (Gao et al. 2019) and could be explained by the competitiveness of unreduced pollen during certation associated with the masking of gametophytic mutational load (Husband 2016). In this scenario, breeders interested in interploidy breeding may benefit from selecting clones bearing a single QTL for unreduced pollen production allowing for interploidy crosses while maintaining fertility at the diploid level.

### Evolutionary relevance of unreduced gametes

Besides being a useful breeding tool, unreduced gametes are a precursor to significant evolutionary innovations such as polyploidy and apomixis (Bicknell and Koltunow 2004; Tayalé and Parisod 2013). Although the role of genetic mutations on unreduced pollen formation was demonstrated in *A. thaliana* (De Storme and Geelen 2013), little is known about the natural genetic variation underlying this trait (Kreiner et al. 2017a). To our knowledge, the candidate alleles *StJR1.t1* and *StJR2.2* are the first natural alleles known to be associated with unreduced pollen production. Kreiner et al. (2017b) extensively studied the rate of unreduced pollen formation across natural populations of *Brassicaceae* species and noted the presence of rare individuals with high level of unreduced pollen production. The rare occurrence of extreme unreduced pollen producers could be explained by the additivity of recessive mutations such as *q2nP_CE_7* and *q2nP_CE_12* identified in our mapping population.

Mutations leading to unreduced pollen formation can be considered maladaptive as they are expected to reduce male fitness in natural diploid populations. As such, they are likely maintained in a dynamic equilibrium between the rate of mutation and the efficacy of purifying selection (Kreiner et al. 2017b). In vegetatively propagated species, purifying selection on sexual processes is relaxed and can allow the maintenance of unreduced pollen mutations at a higher rate (Kreiner et al. 2017a). The ubiquity of unreduced pollen in tuber-bearing *Solanum*, as reported by Watanabe and Peloquin (1989), may be explained by this phenomenon. Those unreduced gametes have played a major role in shaping the tetraploid genome of cultivated potato. Firstly, tetraploid cultivars are likely to be derived from the sexual polyploidisation of early diploid landraces (Hardigan et al. 2017). Secondly, unreduced gametes facilitate interploidy and interspecific gene flow and can therefore account for the prevalent wild *Solanum* introgressions in tetraploid cultivars (Hardigan et al. 2017; Hoopes et al. 2022). Although our candidate alleles *StJR1.t1* and *StJR2.2* offer a molecular basis for those important biological processes, it would be overly simplistic to assume that these two alleles alone comprehensively explain these phenomena. The combination of high mutational load with the multitude of genes involved in meiosis should serve as a reminder that the genetic basis of unreduced pollen production in potato and its wild relatives remains a complex subject. As such, this study may only scratch the surface of the natural variation underlying this trait, leaving room for the discovery of additional mutant alleles in future research.

### Supplementary Information

Below is the link to the electronic supplementary material.

Supplementary file1 (PDF 11448 KB)

Supplementary file2 (IJM 1 KB)

Supplementary file3 (XLSX 31 KB)

## Data Availability

PotatoMASH fastq files for population FRW19-112 are available in the BioProject database under accession number PRJNA858449, while those for populations IVP16-560 and CE-XW are available under the accession number PRJEB65939. Whole genome sequencing fastq files of the parental clones described in this study can be found using the following accession numbers: PRJEB2504 for clone RH89-039-16, PRJEB36551 for clone IVP10-281-1, PRJEB65939 for clones IVP92-057-3 and IVP92-030-14, and PRJEB56778 for clones C and E. The phenotyping data, genotyping data, and all the R code to reproduce the results and figures of this article are available at https://doi.org/10.6084/m9.figshare.24119484.
