# Supplementary material for: Identification of two mutant JASON-RELATED genes associated with unreduced pollen production in potato
Source: Theor Appl Genet. 2024 Mar 12;137(4):79. doi: 10.1007/s00122-024-04563-7 (PMC10933213; doi:10.1007/s00122-024-04563-7)
Supplement: Supplementary file 1 — Supplementary file1 (PDF 11448 KB) [file 122_2024_4563_MOESM1_ESM.pdf]

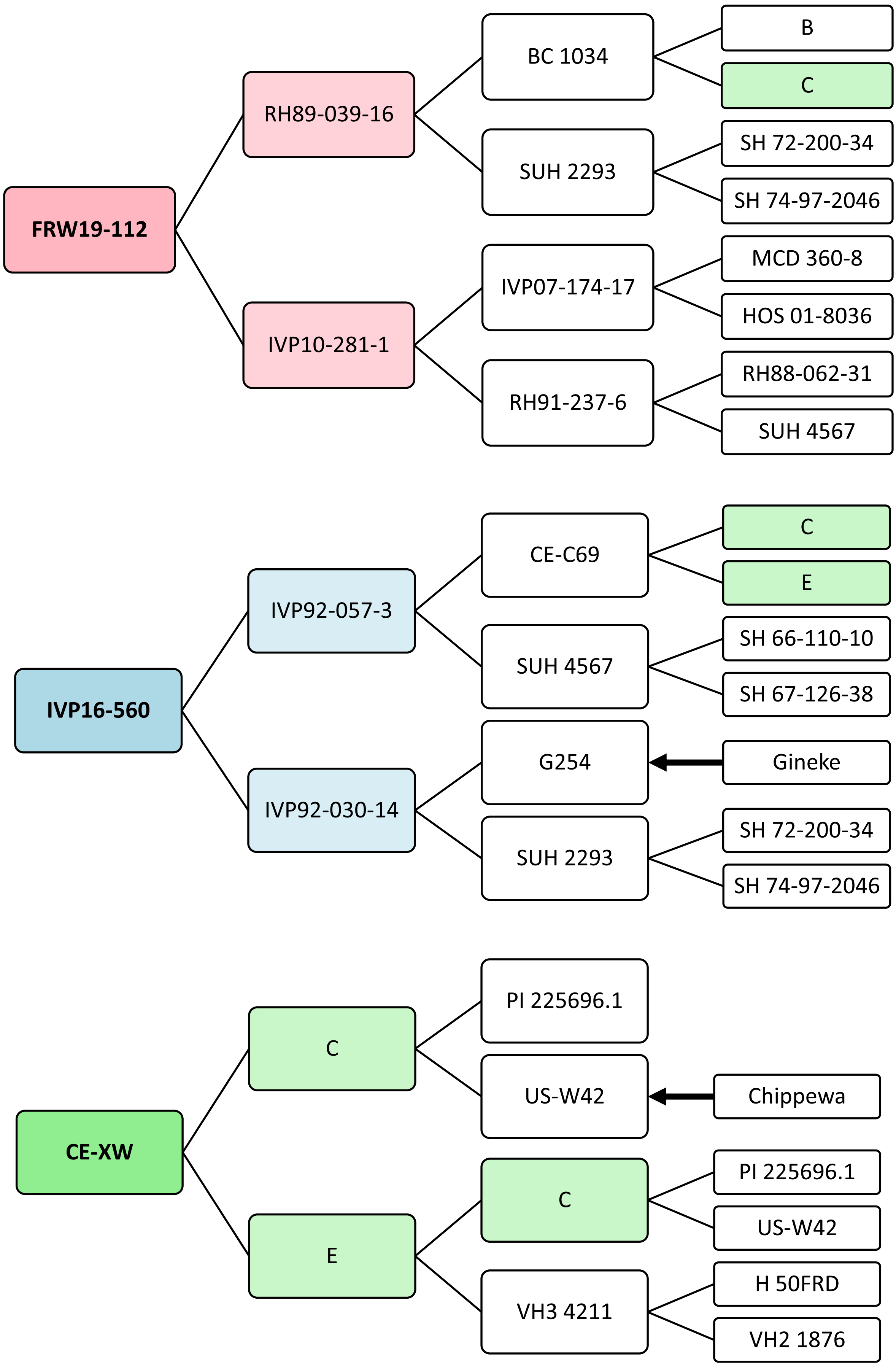

Figure S1: Pedigrees of three bi-parental diploid potato populations. Parental clones of populations FRW19-112, IVP16-560 and CE-XW are highlighted in pink, blue and green respectively. Bold arrows represent dihaploidisation.

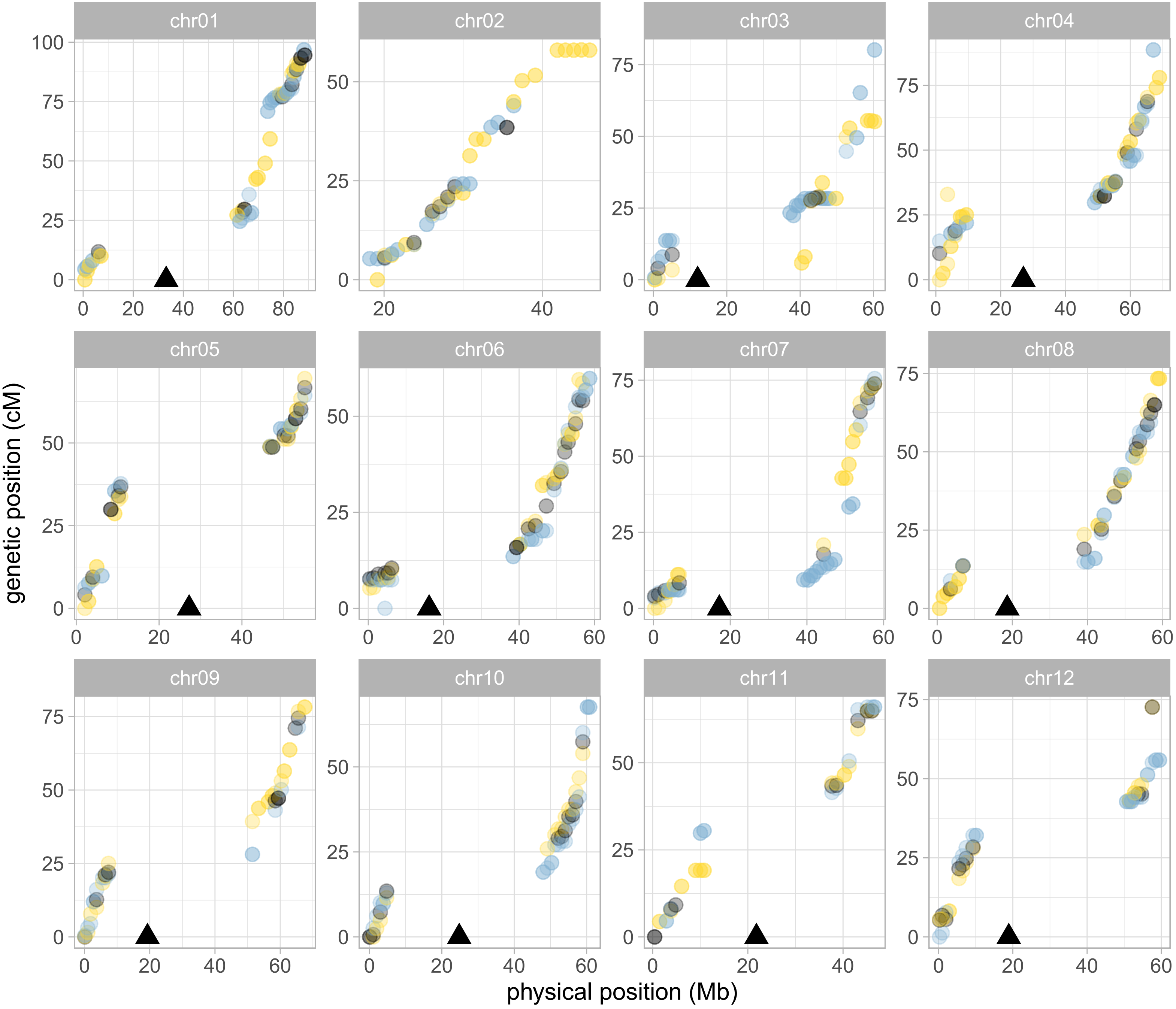

Figure S2: Marey maps in which genetic distance (cM) is plotted over physical distance (Mb). RH89−039−16 markers are displayed in blue, IVP10−281−1 markers in yellow and shared markers in black. Triangles indicate the position of CENH3−binding domains on DM v6.1.

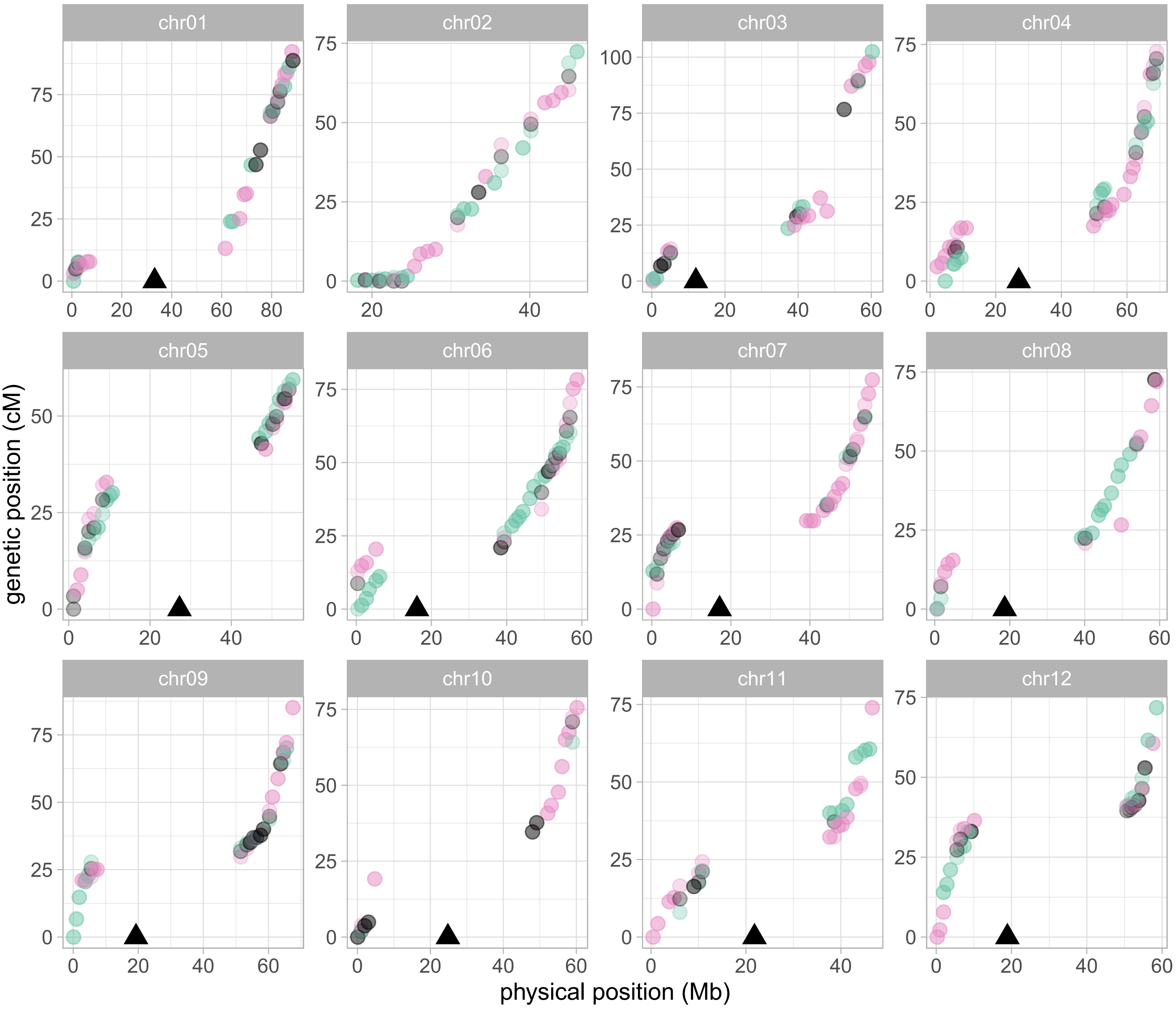

Figure S3: Marey maps in which genetic distance (cM) is plotted over physical distance (Mb). IVP92−057−3 markers are displayed in pink, IVP92−030−14 markers in green and shared markers in black. Triangles indicate the position of CENH3−binding domains on DM v6.1.

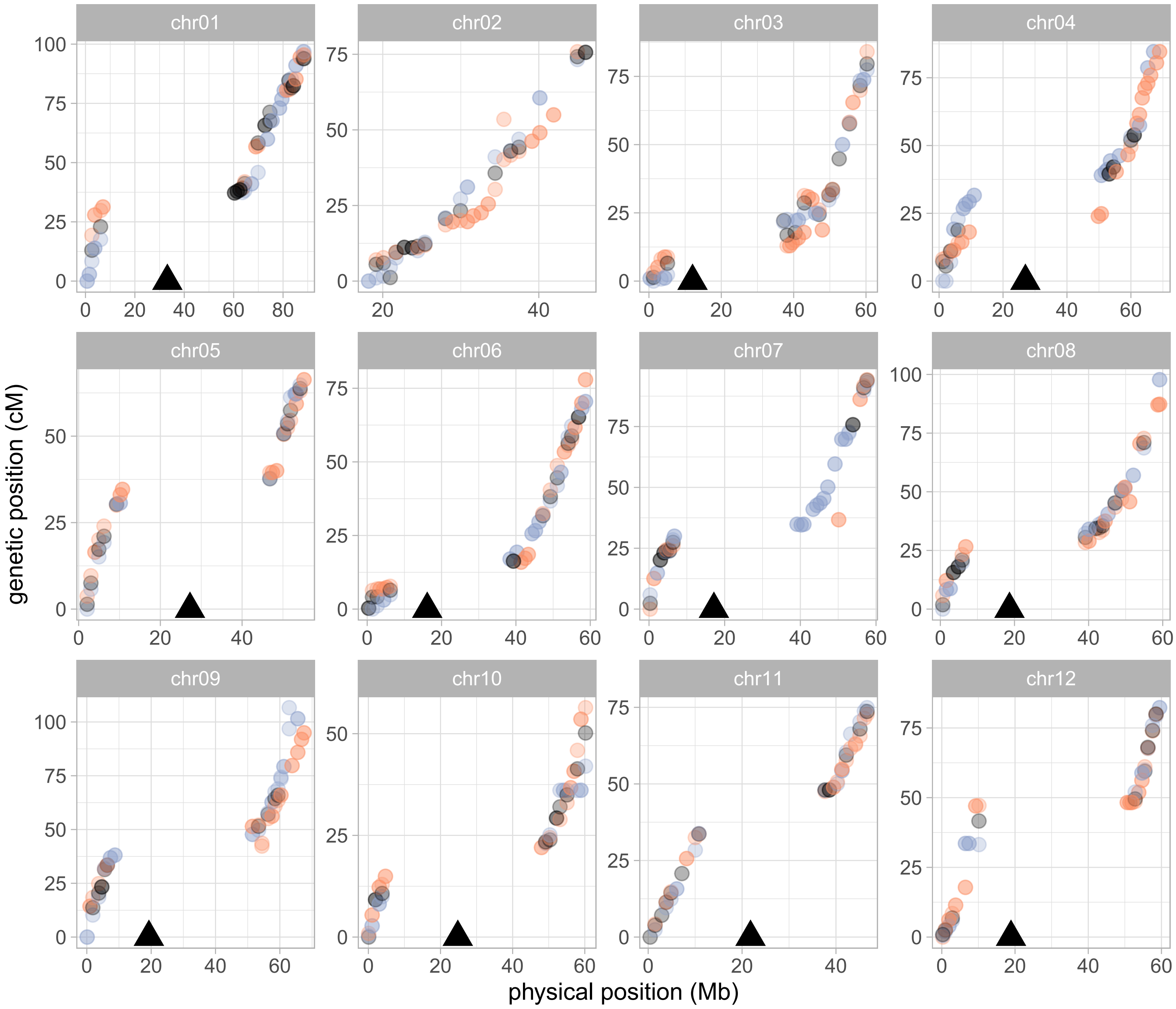

Figure S4: Marey maps in which genetic distance (cM) is plotted over physical distance (Mb). C markers are displayed in orange, E markers in purple and shared markers in black. Triangles indicate the position of CENH3−binding domains on DM v6.1.

(a)

Clone	pachytene	diplotene	diakinesis	metaphase I	anaphase I	telophase I	interkinesis	prometaphase II	metaphase II	anaphase II	telophase II	tetrads	dyads	triads	unbal. products
IVP10-281-1	6	83	104	51	1	5	0	0	0	0	0	0	0	0	0
	4	2	27	80	6	11	112	8	0	0	0	0	0	0	0
	0	0	0	0	0	0	14	59	14	4	36	121	0	2	0
	0	0	0	0	0	0	14	16	8	1	13	194	0	4	0
	0	0	0	0	0	0	0	0	0	0	5	240	0	5	0
RH89-039-16	23	41	146	40	0	0	0	0	0	0	0	0	0	0	0
	0	0	0	78	8	9	115	21	18	1	0	0	0	0	0
	0	0	0	8	2	0	71	54	35	3	7	25	39	3	3
	0	0	0	0	0	0	24	32	70	7	3	52	56	5	1
	0	0	0	0	0	0	0	0	0	4	2	118	83	4	3
IVP92-030-14	2	24	158	62	4	0	0	0	0	0	0	0	0	0	0
	0	0	8	96	2	26	102	13	3	0	0	0	0	0	0
	0	0	0	116	10	16	87	12	0	0	9	0	0	0	0
	0	0	0	12	2	6	116	68	20	2	21	2	0	1	0
	0	0	0	0	0	0	43	58	17	5	38	89	0	0	0
IVP92-057-3	26	10	72	77	12	7	0	0	0	0	0	0	46	0	0
	25	10	29	66	4	15	25	6	5	0	0	1	15	0	0
	0	0	0	52	11	32	64	12	0	2	0	5	72	0	0
	0	0	0	28	1	1	78	26	29	4	3	30	42	5	3
	0	0	0	10	0	0	20	18	43	15	18	68	38	20	0

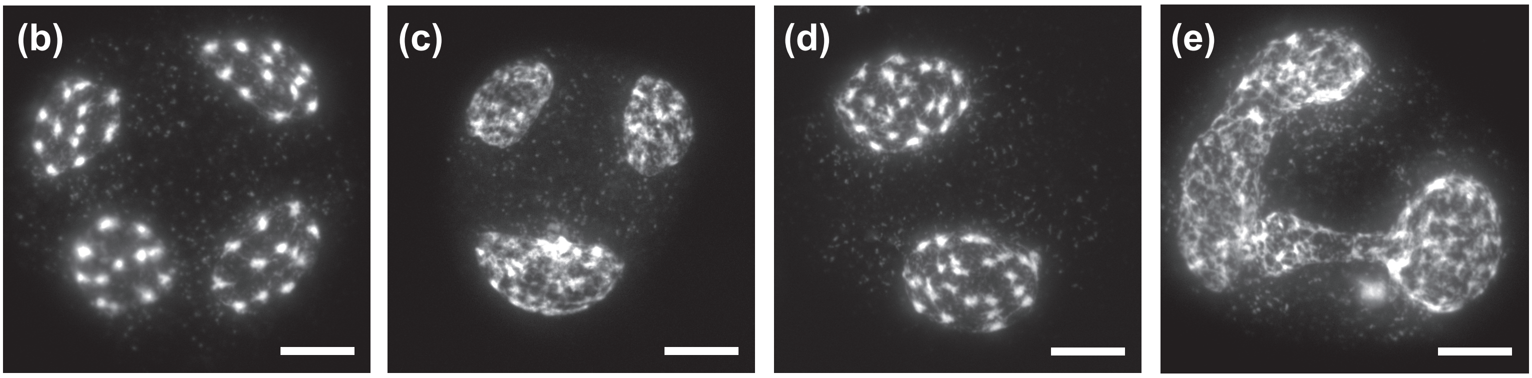

Figure S5: (a) Number of meiocytes at giving meiotic stage observed in the parental clones of population FRW19-119 (in shades of pink) and IVP16-560 (in shades of blue). Each row represent the meiocytes observed in a single anther. (b-e) DAPI-stained chromosome spreads of male meiocytes: tetrad (b), triad (c), dyad (d), nuclei fusion (e). Scale bars = 10 µm.

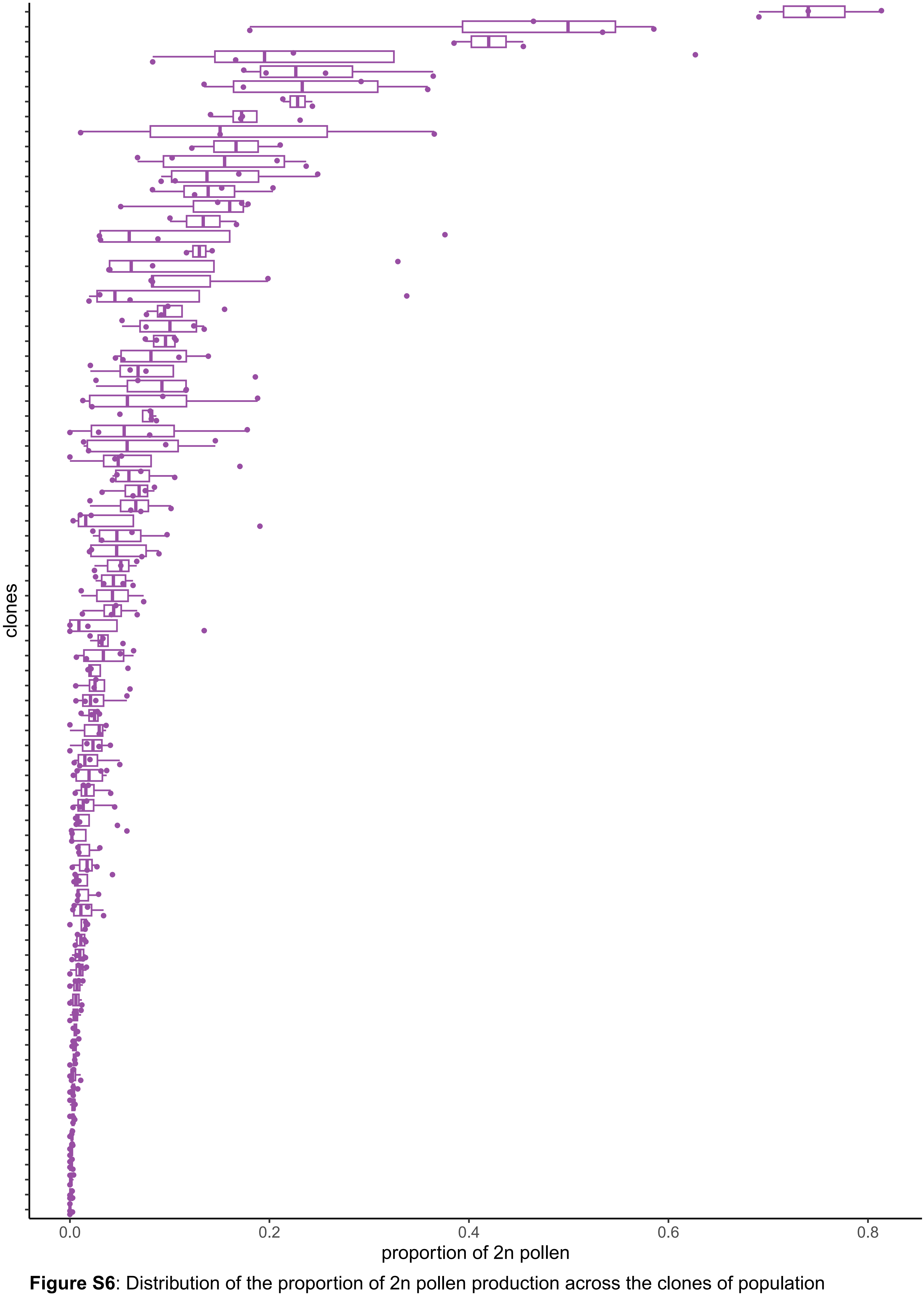

Figure S6: Distribution of the proportion of 2n pollen production across the clones of population FRW19−112.

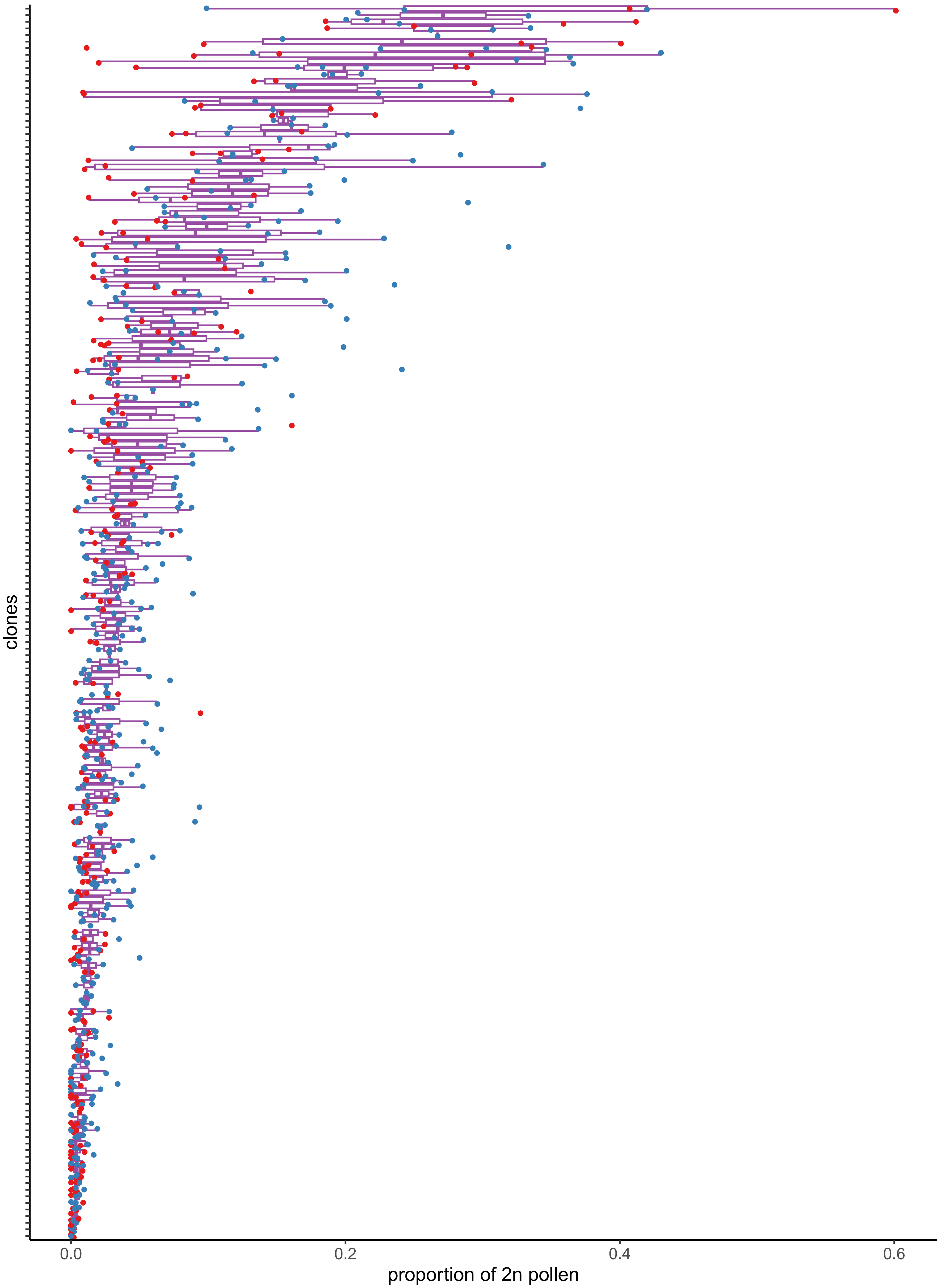

Figure S7: Distribution of the proportion of 2n pollen production across the clones of population IVP16−560. Observations from 2019 and 2020 are shown in red and blue respectively.

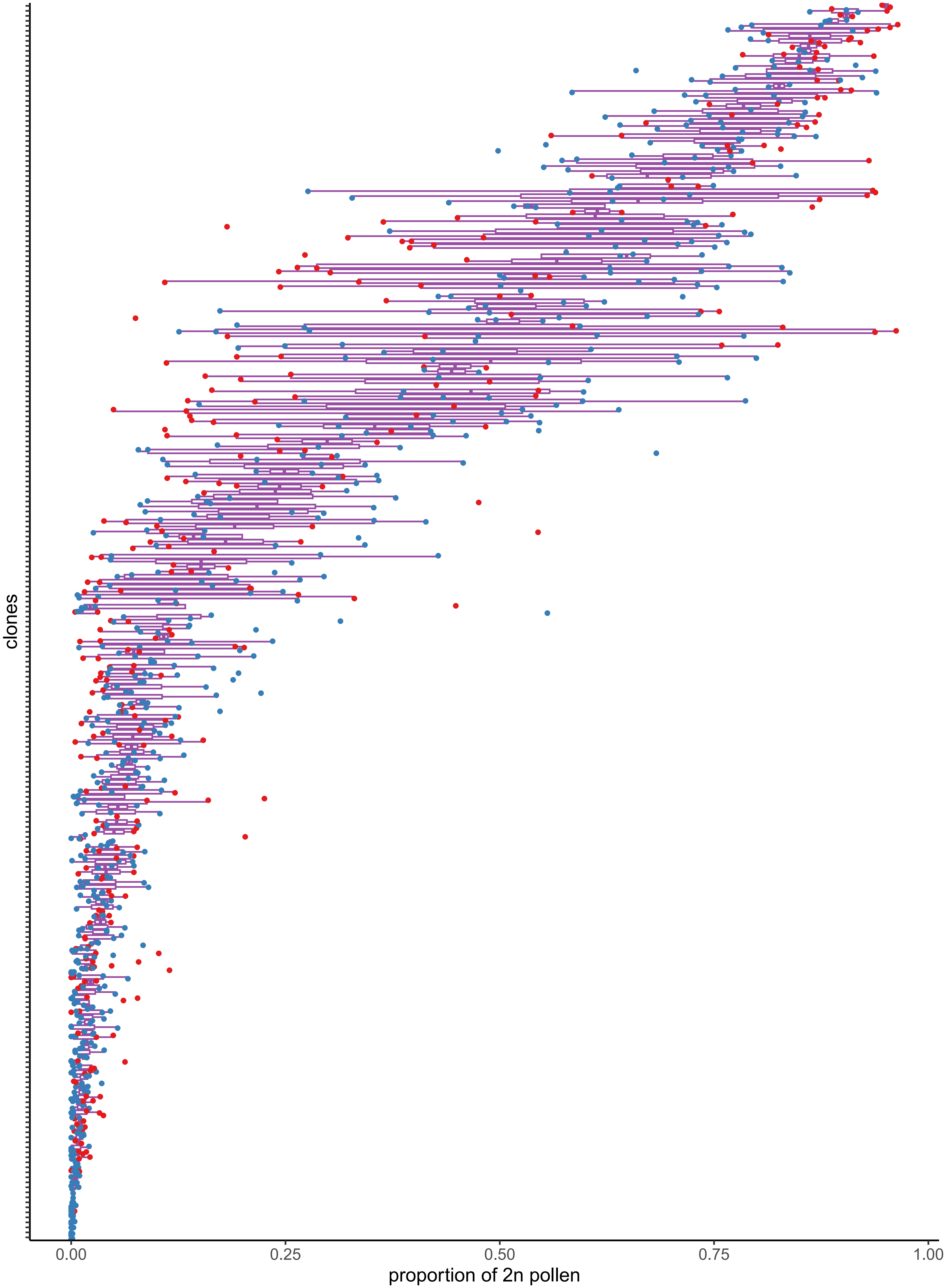

Figure S8: Distribution of the proportion of 2n pollen production across the clones of population CE−XW. Observations from 2020 and 2022 are shown in red and blue respectively.

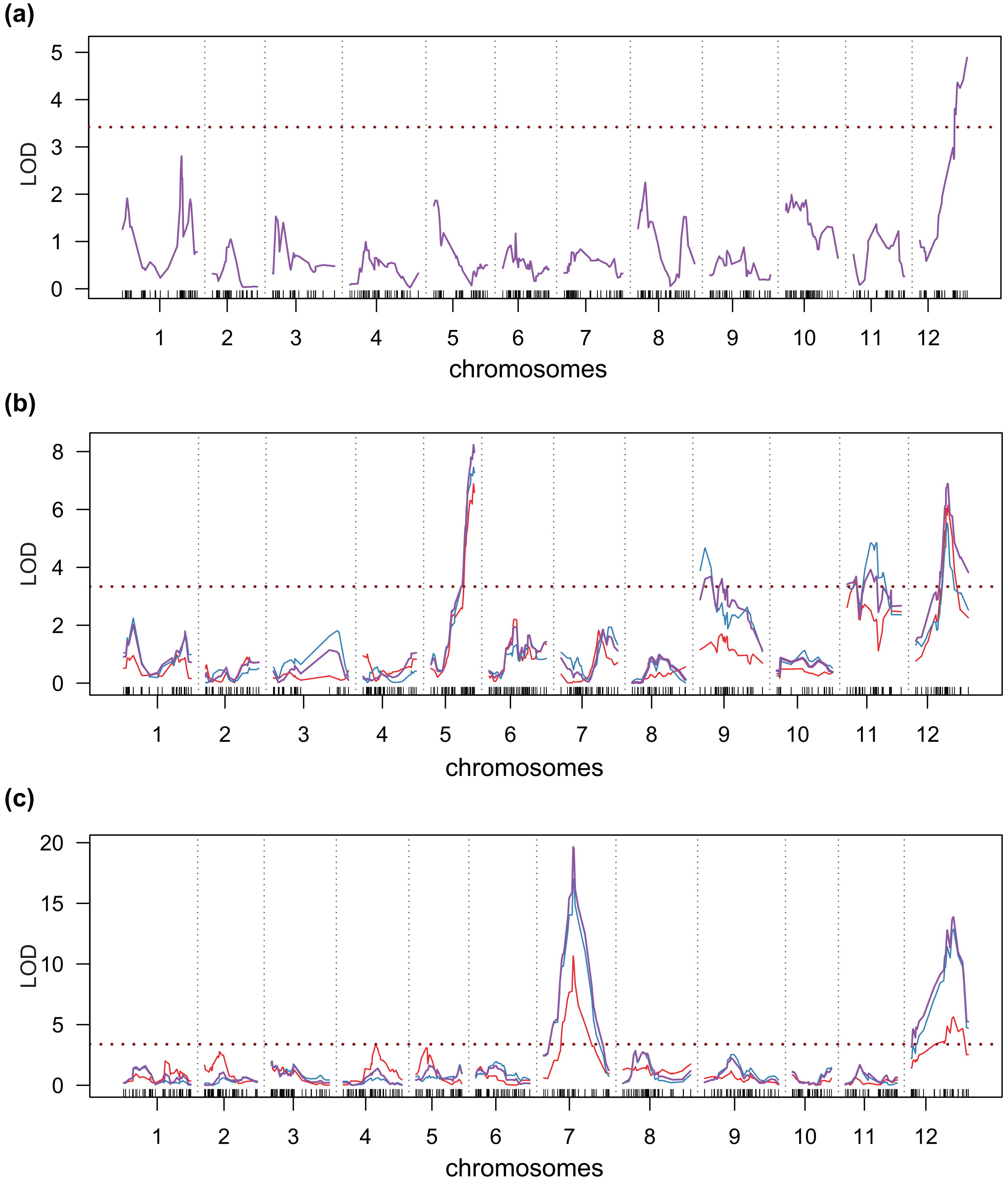

Figure S9: LOD profiles of QTL scan for genotypic BLUPs in FRW19-112 (a), IVP16-560 (b) and CE-XW (c). Significance thresholds, as determined by permutation tests (N = 1000, α = 0.05), are shown as dashed red lines (data were re-scaled so that these overlap). For IVP16-560 (b) and CE-XW (c), data collected the first and second year are shown in red and blue respectively while the combined year data are shown in purple.

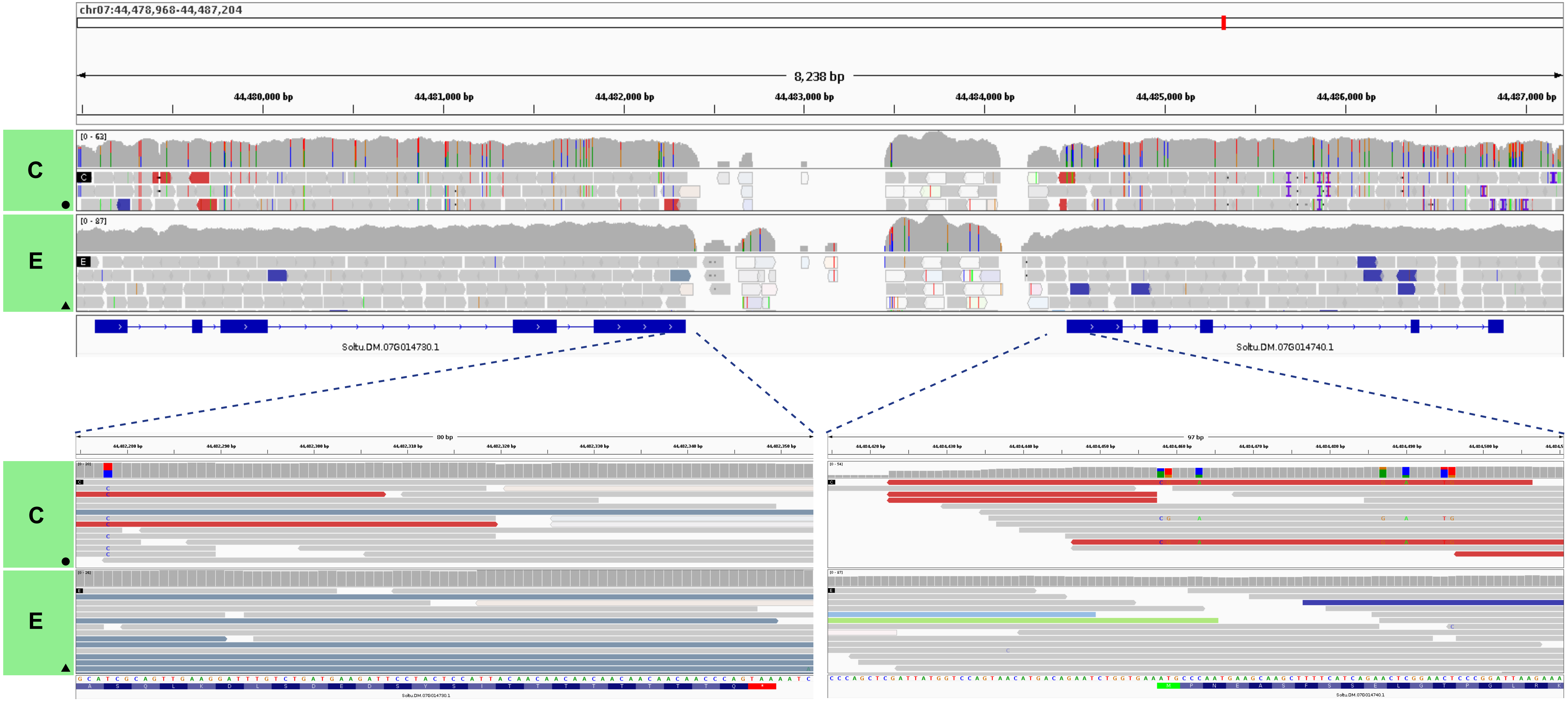

Figure S10: IGV view of the short reads of clone C and E aligned against the reference genome DM v6.1 in the region of *StJR1.t1*. *StJR1.t1* is annotated as two genes due to a 2,103 bp intertion. While clone E is homozygous for *StJR1.t1*, clone C is heterozygous and display pair-end reads coloured in red with an insert size of more than 2,000 bp.

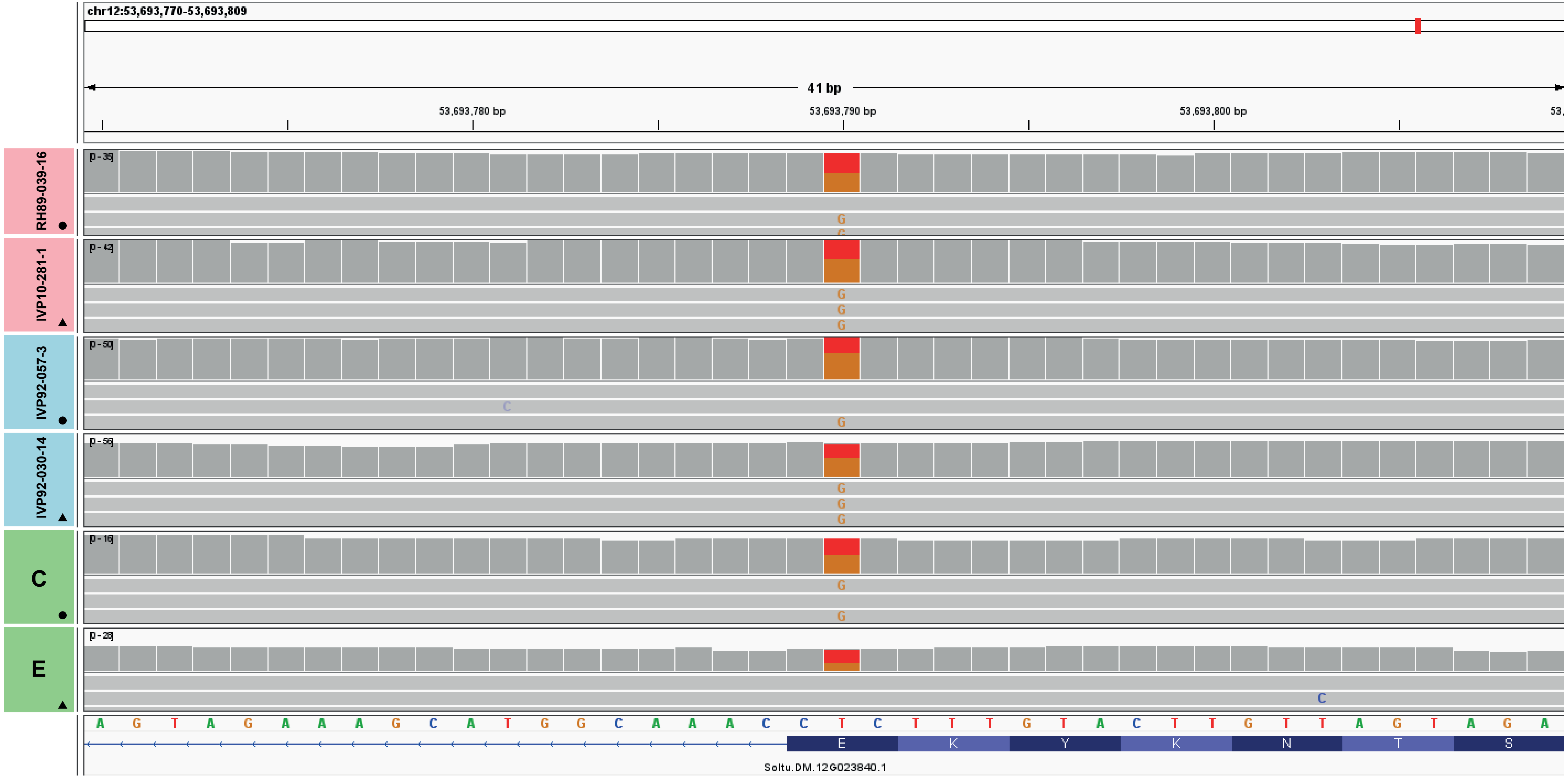

Figure S11: IGV view of the short reads of clone RH89-039-16, IVP10-281-1, IVP92-057-3, IVP92-030-14, C and E aligned against the reference genome DM v6.1. This view focus on the 5th exon of *StJR2* where a T>G missense mutation leading to a Glu>Ala substitution in the highly conserved C-terminal domain of StJR2 is found in all 6 clones.
